# Higher serum levels of periostin and the risk of exacerbations in moderate asthmatics

**DOI:** 10.1186/s40733-015-0019-x

**Published:** 2016-01-04

**Authors:** N. Scichilone, C. Crimi, A. Benfante, S. Battaglia, M. Iemmolo, M. Spatafora, N. Crimi

**Affiliations:** 1 Dipartimento Biomedico di Medicina Interna e Specialistica (Di.Bi.M.I.S.), University of Palermo, Palermo, Italy; 2 Euro-Mediterranean Institute of Science and Technology, Palermo, Italy; 3 Department of Clinical and Molecular Biomedicine, University of Catania, Catania, Italy

**Keywords:** Serum biomarker, Asthma, Periostin, Exacerbation, Airway inflammation

## Abstract

**Background:**

In asthma, exacerbations and poor disease control are linked to airway allergic inflammation. Serum periostin has been proposed as a systemic biomarker of eosinophilic inflammation. This pilot study aims at evaluating whether in patients with moderate asthma, higher baseline levels of serum periostin are associated with a greater risk of exacerbation.

**Methods:**

Fifteen outpatients with moderate allergic asthma were recruited. Serum concentrations of periostin were assessed (ELISA) at baseline, and the frequency of asthma exacerbations was recorded during a one-year follow-up.

**Results:**

Patients (M/F: 10/5, mean age of 47.6 ± 11.0 years) had mean ACQ score of 5.5 ± 4.2 and FEV_1_%pred of 81.9 ± 21.7 %. Baseline serum levels of periostin did not correlate with lung function parameters, nor with the ACQ score (p ≥0.05 for all analyses). Five subjects (33 % of the study group) reported one or more exacerbations during the following year. Baseline serum levels of periostin were significantly higher in subjects who experienced one or more exacerbations during the one year period of follow-up, compared with subjects with no exacerbations: median serum periostin level was 4047 ng/ml (range: 2231 to 4889 ng/ml) and 222 ng/ml (range 28.2 to 1631 ng/ml) respectively; *p* = 0.001.

**Conclusion:**

The findings of the present pilot study could form the basis for the design of larger studies aiming at developing strategies to identify asthmatic patients at risk for exacerbations.

## Background

The long-term goals in asthma management are the optimal control of respiratory symptoms and the risk reduction of future exacerbations [1]. Exacerbations are linked to the greater decline in lung function and to airway inflammation expressed by an increase of eosinophil count in the sputum and of exhaled nitric oxide [2]. These markers of eosinophilic inflammation are also related with poor control of asthma [3]. Periostin proteins in peripheral blood correlate with airway eosinophilic inflammation, and have therefore the potential to be used as a biomarker to select patients for asthma therapies targeting TH2 inflammation [4]. In this regard, serum periostin has been found to correlate with the increase of eosinophil count in peripheral blood, in the induced sputum and with asthma control in patients with severe and uncontrolled asthma [4]. Recently, Kenemitsu and colleagues [5] found that higher serum levels of periostin were associated with greater decline in lung function in asthmatics.

Periostin has been used as a biomarker to predict the response to TH2-target therapy with humanized monoclonal antibody to IL-13 (lebrikizumab) in uncontrolled asthma patients: patients with high level of serum periostin at baseline showed a greater improvement with lebrikizumab treatment, in comparison with patients with low level of serum periostin [6]. The post-hoc analysis of the “Extra Study” [7] in uncontrolled severe asthmatics treated with omalizumab demonstrated a reduction in exacerbations by 30 % in patients with high levels of periostin. These data support the hypothesis that periostin could predict the response to treatment in unstable patients with TH2 driven phenotype.

The aim of this study is to evaluate, in a pilot fashion, whether patients with moderate asthma and higher baseline levels of serum periostin show a greater risk of exacerbation during a year of follow-up.

## Methods

### Subjects

Unselected consecutive individuals who fulfilled the diagnostic criteria for asthma were enrolled [1]. Fifteen asthmatics with moderate asthma were recruited at the outpatient clinic for respiratory allergic diseases of the University of Palermo, Italy. Current smoker patients, or ex smokers defined as smoking at least ten pack-year, together with those affected by respiratory diseases other than asthma were excluded from the study. To enter the study, all subjects had to be under regular inhaled treatment with beta-2 adrenergic long-acting bronchodilators and corticosteroids (LABA/ICS in fixed combinations) for a period of at least six months at the time of enrollment. Patients were not different in terms of history of exacerbations in the year before the enrollment. The local Ethics Committee approved the protocol and written informed consents were obtained.

### Study design

The study had a duration of 52 weeks. After giving their consent, all enrolled patients underwent an initial assessment (Visit 1) during which clinical and lung functional evaluations were performed. Peripheral blood was then collected for the periostin concentration measurements. All patients were then given the same LABA/ICS formulation at the study entry and were followed for one year, at three-month intervals. At each visit, compliance with treatment was also assessed by the clinical investigators by asking the patients to return the empty devices; the correct use of the device was also assessed. Unscheduled visits were allowed when symptoms worsened to identify any exacerbation, which was treated with the use of oral corticosteroids; the frequency of asthma exacerbations was then recorded at three-month intervals. Salbutamol as rescue medication, as well as anti-histamines during exposure to allergens causing nasal and/or ocular symptoms, were allowed during the study period. No other comorbid conditions or non respiratory medications were present at the time of the study.

### Clinical and functional assessments

Demographic and clinical data included age, sex, duration of disease, current treatment and the evaluation of the atopic condition. The level of asthma control was evaluated by the asthma control questionnaire (ACQ). Exacerbation was defined as any acute worsening of respiratory symptoms, such as shortness of breath, cough, wheezing or chest tightness, which required changes in treatment (i.e. use of oral corticosteroids), according to the GINA guidelines [1]. Functional assessment included measures of static and dynamic lung volumes, and was performed using a fully computerized Stead-Wells Water Seal Spirometer (Baires System; Biomedin; Padua, Italy), using the helium dilution method. Measurements were made in accordance to the European Thoracic Society standardization of lung volume measurements [8]. FEV_1_ %pred (forced expiratory volume in the 1st second as % of predicted) and FVC %pred (forced vital capacity as % of predicted), as well as TLC %pred (total lung capacity as % of predicted), were calculated. All patients underwent a skin prick testing with a standard panel of airborne allergens (*grasses, pellitory, ragweed, birch, cypressus, olive, dermatophagoides pteronyssinus and farinae, cat and dog epithelia, alternaria tenuis, aspergillus fumigatus*). The test was considered positive if the mean wheal diameter was 3 mm or larger after the subtraction of the mean wheal of the negative control.

### Periostin serum level analysis

Human periostin was dosed in serum samples. The assay was performed by ELISA kit Human Periostin/OSF 2 of Aviscera Bioscience. The kit contains the necessary components required for the quantitative measurement of recombinant and natural human periostin/OSF 2 in biological samples employing sandwich ELISA format. The plate is pre-coated with antibody specific for Periostin able to capture the periostin in the standard and the samples. The periostin captured was detected by the addition of a further periostin specific biotinylated antibody and streptavidin-HPR with its specific substrate. It follows the emission of colorimetric signal directly proportional to the concentration of periostin in the sample. This signal was then measured by microplate spectrophotometer at 450 nm. A standard curve was established by plotting the log of the known concentrations of the standard dilution (x axis) versus the log of this corresponding O.D. (y-axis) and the log-log curve was generated for the calculation of periostin (ng/ml) in serum samples. The detection limit of the ELISA assay used to assess periostin levels was 2.0 ng/ml. The measurements were performed blindly by one of the authors (MI), and all investigators were never aware of the periostin levels over the year of observation.

### Statistical analysis

Statistical analysis was carried out using SPSS 21 and GrapfPad Prism 6.0 for Windows. Normally distributed data are reported as mean ± standard deviation (SD) and skewed data by median and range. Comparisons were performer by parametric (Student’s t test) or non-parametric (Mann-Whitney U tests) tests, as appropriate. The relationships between variables are expressed as Spearman’s rank correlation coefficient. For each test, a p value of less than 0.05 was taken as the threshold of statistical significance.

## Results

A total of 15 moderate asthmatics (M/F: 10/5) were studied. Patients had mean age of 47.6 ± 11.0 years, and mean ACQ score of 5.5 ± 4.2. Lung function characteristics of the study subjects are presented in Table 1. All subjects were skin test positive to at least one aeroallergen, and none was a current smoker (five subjects were former smokers). In particular, 9 subjects were positive to one aeroallergen (*Dermatophagoides* or *Parietaria*), whereas the remaining 6 individuals showed multiple allergen sensitizations.

**Table 1:** Demographic and lung function characteristics of the study subjects

#	Gender	Age	Smoke	FEV1%	FVC%	FEV1/FVC	RV%	TLC%	RV/TLC%	FRC%
1	F	37	n	108	133	70,6	83	116	72	92
2	M	64	n	109	102	78,2	88	93	90	85
3	M	33	n	77	78	79,4	79	77	99	54
4	M	44	f	91	105	70,4	77	96	79	70
5	F	55	n	83	112	63,3	86	97	86	112
6	M	35	f	54	88	49,6	96	90	108	93
7	M	31	f	73	95	61,4	99	96	102	97
8	F	44	n	107	130	71,1	106	118	91	83
9	M	48	n	62	87	55,5	138	101	127	112
10	M	61	n	61	76	61,2	107	86	126	84
11	F	41	f	86	91	81,9	96	90	106	75
12	F	46	n	110	108	88,1	98	100	99	77
13	M	62	f	100	94	81,2	88	89	94	81
14	M	58	n	59	95	48,0	148	114	135	116
15	M	55	n	49	73	52,3	114	86	129	108
Mean (SD)	-	47.6 (11.0)	-	81.9 (21.7)	97.8 (17.8)	67.5 (12.7)	100.2 (20.4)	96.6 (11.8)	102.9 (19.1)	89.3 (17.5)

*Abbreviations*: for Gender: *M* male, *F* female. For Smoke: *f* former smoker, *n* never smoker

FEV1%: forced expiratory volume at first second, % predicted, FVC%: forced vital capacity, % predicted, RV%: residual volume, % predicted, TLC%: total lung capacity, % predicted, FRC%: functional residual capacity, % predicted

At baseline, median values of serum periostin were 773 ng/ml (range: 28-4889 ng/ml). No statistically significant correlations were detected between baseline serum levels of periostin and lung function parameters; similarly, serum periostin was not associated with the ACQ score (p ≥0.05 for all analyses). Furthermore, serum periostin levels did not show any association with the allergic pattern (mono vs. polysensitized subjects) or age at onset of asthma (data not shown). In the following year, 5 subjects (33 % of the study group) reported one or more asthma exacerbations (4 individuals with one exacerbation and 1 individual with two exacerbations), whereas the other 10 subjects did not experience any exacerbation in the follow-up period. The levels of periostin did not predict the time to the first exacerbation, nor did they correlate with the rate of exacerbations. All exacerbations were treated with a course of oral corticosteroids, and disappeared within one week of treatment, with no need to be admitted to hospital.

**Fig. 1:**
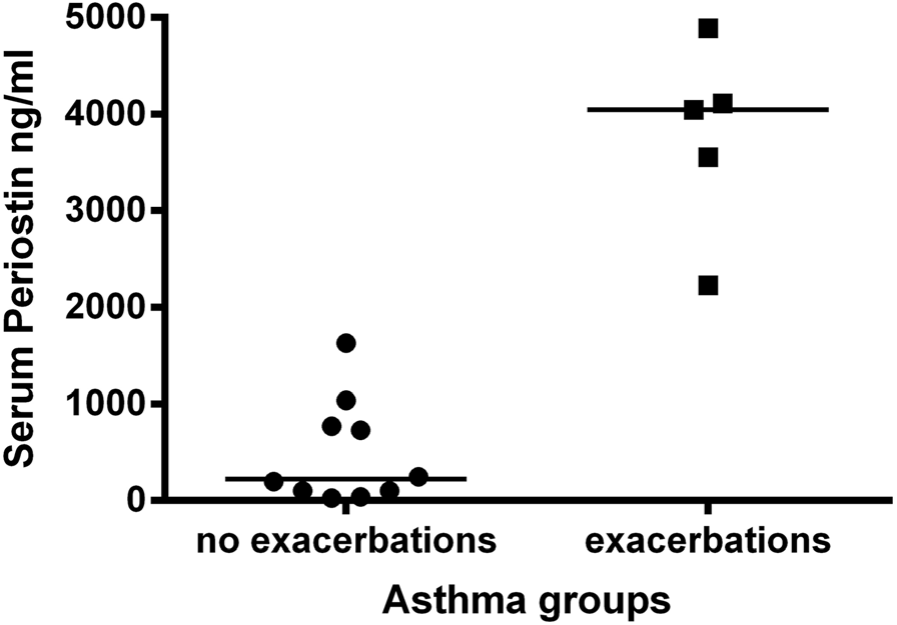
Differences in serum periostin level between asthmatic subjects with no exacerbations and those with one or more exacerbations. Horizontal bars represent median

Baseline serum levels of periostin were significantly higher in subjects who experienced one or more exacerbations during the follow-up, compared with those with no exacerbations: median serum periostin levels were 4047.7 ng/ml (range: 2231.7 to 4889.0 ng/ml) and 222.0 ng/ml (range 28.2 to 1631.4 ng/ml), respectively; *p* = 0.001 (Fig. 1).

## Discussion

The main finding of this explorative study is that serum periostin concentrations are increased in moderate asthmatics with unstable disease. Our observations advocate for larger confirmatory studies whether serum periostin as a predictive biomarker for greater risk of exacerbations.

The novelty of the current study is that high serum periostin concentrations appears to predict acute asthma exacerbations in the following year, providing evidence for the potential association between persistent T_H_2- or IL-13–driven inflammation and greater risk for loss of asthma control. This is further emphasized by a recent study by Song et al. [9], who showed a strong relationship between serum concentrations of periostin and airway hyperresponsiveness (AHR) to methacholine and mannitol in asthmatic children. Moreover, Kenemitsu and colleagues [5] showed that, when patients were stratified into two groups according to their serum periostin levels, high serum periostin (≥95 ng/mL) was also associated with greater decline in FEV_1_. Recently, Nagasaki et al. [10] found that in patients with elevated FeNO, the identification of high serum periostin levels allowed to identify patients with faster FEV_1_ decline and risk of asthma exacerbation, despite high doses of ICS. These data are consistent with the notion that periostin is a strong determinant of future risk in asthma. However, contradictory evidences exist: Gordon et al. [11] explored the biological role of periostin in asthma using a mouse model and periostin deficient mice and, surprisingly, found that periostin deficient mice have increased AHR and markedly increased serum IgE levels following repeated intranasal challenge with *Aspergillus fumigatus* antigen, compared to wild-type mice controls. This led the Authors to propose a protective role for epithelial cell-derived periostin, probably mediated by TGF-beta-induced differentiation of T regulatory cells.

Since ICS treatment reduces airway periostin expression [12] and airway eosinophilia [13], high levels of serum periostin represent a marker of persistent airway eosinophilia despite ICS treatment in patients with uncontrolled asthma [4]. It is possible to speculate that our study subjects who experienced exacerbations were among those in whom inhaled treatment did not suppress the eosinophilic inflammation. However, recent evidence demonstrated that serum periostin is not able to distinguish eosinophilic from non-eosinophilic airway inflammation, whereas blood eosinophils had the highest accuracy in the identification of sputum eosinophilia in asthma [14]. The lack of measurements of sputum or blood eosinophils in the current study limits any inference in this respect.

The present study has several limitations. Serum periostin was measured only once at enrollment, consequently there are no data that may allow for the assessment of intra-patient variability. However, low intra-patient variability for serum periostin has been already demonstrated [6]. The small number of recruited subjects may have affected the findings of the study. Due to the explorative nature of this study, results need to be confirmed in a larger sample of patients; however, the prospective nature and the design of the present study allowed a precise collection of each exacerbation event, thus providing reliable data for the analysis. Finally, the apparent discrepancy of the levels of serum periostin between the current study and other observations can be likely attributed to the different methodologies used. Although much higher than those measured elsewhere, the periostin levels were still able to discriminate between subjects with recurrent exacerbations and those in stable conditions.

## Conclusions

In conclusion, periostin could be envision as a novel biomarker to better characterize asthmatic subjects with higher risk of exacerbations. The findings of the present pilot study could form the basis for the design of larger studies aiming at developing strategies to identify patients at risk for exacerbations.

## Abbreviations

ACQ: asthma control questionnaire
FeNO: fractional exhaled nitric oxide
FEV_1_: forced expiratory volume in the 1st second
FVC: forced vital capacity
ICS: inhaled corticosteroids
IgE: immunoglobulin E
IL-13: interleukin 13
LABA: long-acting bronchodilators beta-2 adrenergic
SD: standard deviation
TGF beta: transforming growth factor-beta
TH2: T helper cell 2
TLC: total lung capacity
